# Difference in gating and doping effects on the band gap in bilayer graphene

**DOI:** 10.1038/s41598-017-11822-9

**Published:** 2017-09-12

**Authors:** Takaki Uchiyama, Hidenori Goto, Hidehiko Akiyoshi, Ritsuko Eguchi, Takao Nishikawa, Hiroshi Osada, Yoshihiro Kubozono

**Affiliations:** 1 Research Institute for Interdisciplinary Science, Okayama University, Okayama, 700-8530 Japan; 2 Hanamaki Satellite, Research Center for Industrial Science, Iwate University, Iwate, 025-0312 Japan; 3 Faculty of Science and Engineering, Iwate University, Iwate, 020-8551 Japan

## Abstract

A band gap is opened in bilayer graphene (BLG) by applying an electric field perpendicular to the layer, which offers versatility and controllability in graphene-based electronics. The presence of the band gap has been confirmed using double-gated BLG devices in which positive and negative gate voltages are applied to each side of BLG. An alternative method to induce the electric field is electron and hole doping of each side of BLG using electron-transfer adsorbates. However, the generation of the band gap by carrier doping is still under investigation. Here, we determined whether the electron/hole doping can produce the electric field required to open the band gap by measuring the temperature dependence of conductivity for BLG placed between electron-donor self-assembled monolayers (SAMs) and electron-acceptor molecules. We found that some devices exhibited a band gap and others did not. The potentially irregular and variable structure of SAMs may affect the configuration of the electric field, yielding variable electronic properties. This study demonstrates the essential differences between gating and doping.

## Introduction

Control of carrier density is a key issue when engineering the electronic properties of materials. Various phase transitions, such as insulator-metal^1–4^, insulator-superconductor^5–7^, and nonmagnetic-magnetic^8, 9^ transitions have been caused by tuning the carrier density. Two methods are widely used for this purpose. One is a gating method, which accumulates carriers electrostatically using a field-effect transistor (FET) structure. The other is a doping method, based on electron transfer between dopants and target materials. These two methods are generally believed to work in essentially the same way. Indeed, both gating and doping methods have enabled us to induce superconductivity in SrTiO_3_^10, 11^, MoS_2_^12, 13^, MoSe_2_^14, 15^, and LaOBiS_2_^16, 17^, to name a few.

**Figure 1:**
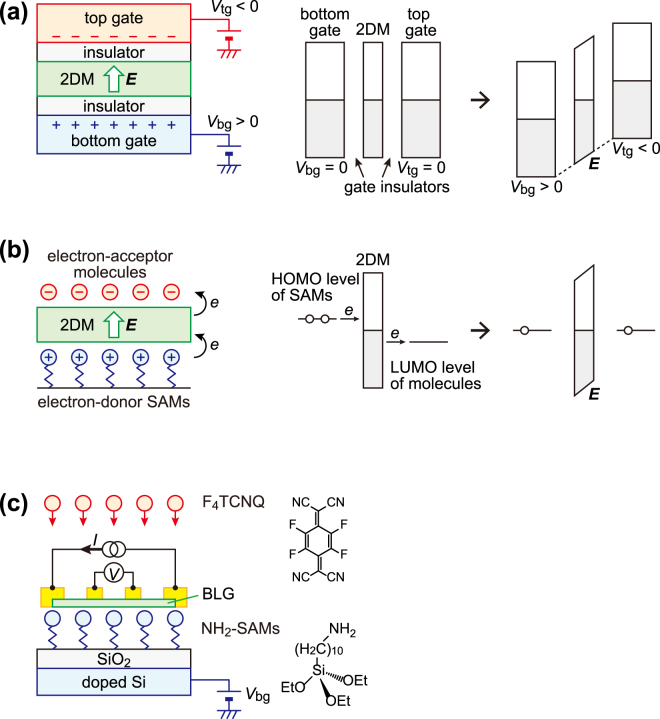
Two methods to generate an electric field. (**a**) Double-gate method; positive bottom- and negative top-gate voltages produce an upward electric field (left). Energy band diagrams of 2DM and two gate electrodes are illustrated before (middle) and after (right) applying gate voltages. The origin of the electric field is the potential difference between two gate electrodes. (**b**) Double-dope method; electron-donor SAMs and electron-acceptor molecules can produce an upward electric field (left). Energy band diagrams of 2DM, SAMs and molecules are illustrated before (middle) and after (right) electron transfer. The origin of the electric field is the Fermi level alignment of 2DM, SAMs and the molecules. (**c**) Schematic of device structure used in this study. BLG is placed on NH_2_-SAMs. Four-terminal conductivity is compared before and after the deposition of F_4_TCNQ molecules. Molecular structures of F_4_TCNQ and NH_2_-SAMs are shown.

A double-gate technique using top and bottom gates extends the controllability of electronic states not only by tuning carrier density but also by producing an electric field. When different voltages, *V*_tg_ and *V*_bg_, are applied to the top and bottom gates, respectively, the potential difference between the two gates produces an electric field (Fig. 1a). This effect is important, especially in two-dimensional layered materials (2DMs) through which the electric field can penetrate. A notable example is bilayer graphene (BLG), which has two quasi-quadratic bands in contact with each other. This zero-gap semiconductor exhibits a band gap when it was subjected to a perpendicular electric field^18^, which was confirmed from transport and optical properties^19–21^ for a double-gated FET structure. The effective band gap estimated from transport has been much smaller than that from optical measurement^19–26^. The origin of the smaller transport gap remains to be clarified, although it can be attributed not only to variable range hopping in gap states^19, 22–24^ but also to one-dimensional conduction through edges^25^ or domain walls^26^.

The double-doping method (or electron/hole doping) is also expected to give the same effect in BLG as the double-gating, although the mechanism that produces the electric field is completely different. In the case of double-doping, the electron transfer between dopants and BLG aligns their Fermi levels, thus inducing the electric field (Fig. 1b). For example, n-doping of the bottom layer and p-doping of the top layer can create an electric field directed from the bottom to the top layer, which will open a band gap in BLG.

The electronic property of doped BLG has been studied in several experiments thus far^27–30^, and the presence of a band gap is confirmed in some reports^27, 28^. An early study with angle-resolved photoemission spectroscopy (ARPES)^27^ showed that a band gap was actually opened in BLG in which the bottom layer was n-doped by a SiC substrate and the top layer was p-doped by deposition of electron acceptor molecules. Transport and infrared (IR) absorption measurements also demonstrated the existence of a band gap in BLG placed between electron-donor self-assembled monolayers (SAMs) and electron-acceptor molecules^28^. On the other hand, the band gap was not indicated in other reports^29, 30^. The *V*_bg_ dependence of conductivity was measured in BLG on which K atoms^29^ or O_2_ molecules^30^ were adsorbed. The top layer of BLG was n-doped by K atoms^29^ or p-doped by O_2_ molecules^30^, and the electric field is expected to be induced between the bottom gate and the adsorbates at the charge neutrality point. But neither study indicated the band gap. Thus, the reported results have been inconsistent.

To gain insight into this inconsistency, we studied whether a band gap in BLG is opened with the doping method by measuring the temperature dependence of its transport property. The emergence of a band gap was unambiguously confirmed by the suppression of carrier density at low temperatures, while previous measurements^27–30^ have been carried out at room temperature.

Figure 1c illustrates the device structure used in this study. BLG is placed between SAMs and adsorbed molecules. The SAMs were prepared from 10-aminodecyltriethoxysilane, called NH_2_-SAMs. This has an end group of NH_2_ which donates an electron to the bottom layer of BLG^31^. For the other side, typical electron acceptor molecules, 2,3,5,6-tetrafluoro-7,7,8,8-tetracyanoquinodimethane (F_4_TCNQ), were deposited to donate holes to the top layer of BLG. The F_4_TCNQ molecule is a strong electron acceptor which accumulates nearly 1 hole per molecule at low coverage^32^. Sandwiched between these molecules, BLG can be influenced by the upwardly directed electric field. The molecular structures of NH_2_-SAMs and F_4_TCNQ are shown in Fig. 1c.

First, NH_2_-SAMs were prepared on SiO_2_ (300-nm thick)/Si substrates by a liquid-phase method (described in detail elsewhere^33^). Graphene flakes were deposited on the NH_2_-SAMs with a cleavage technique^34^, and BLG was identified by optical contrast^35, 36^ and Raman spectroscopy^37^. Electrodes were attached to the flakes in a four-terminal configuration by using electron beam lithography and vacuum evaporation of metals (5-nm thick Cr and 100-nm thick Au). The devices were placed in a cryogenic probe station, and their four-terminal conductivity *σ* was measured as a function of bottom gate voltage *V*_bg_ with decreasing temperature; *σ*(*V*_bg_) refers to the *σ* for *V*_bg_. After the transport measurement, the device was transferred to the other chamber in order to deposit F_4_TCNQ molecules. The deposition was carried out while keeping the substrate at room temperature and the thickness, *t*, was determined with a thickness monitor. Subsequently, the device was returned to the probe station and the temperature dependence of *σ*(*V*_bg_) curve was measured again. The process of transport measurement and molecular deposition was repeated in ultrahigh vacuum under 10^−6^ Pa without exposing the device to air.

## Results

### Temperature dependence of conductivity

**Figure 2:**
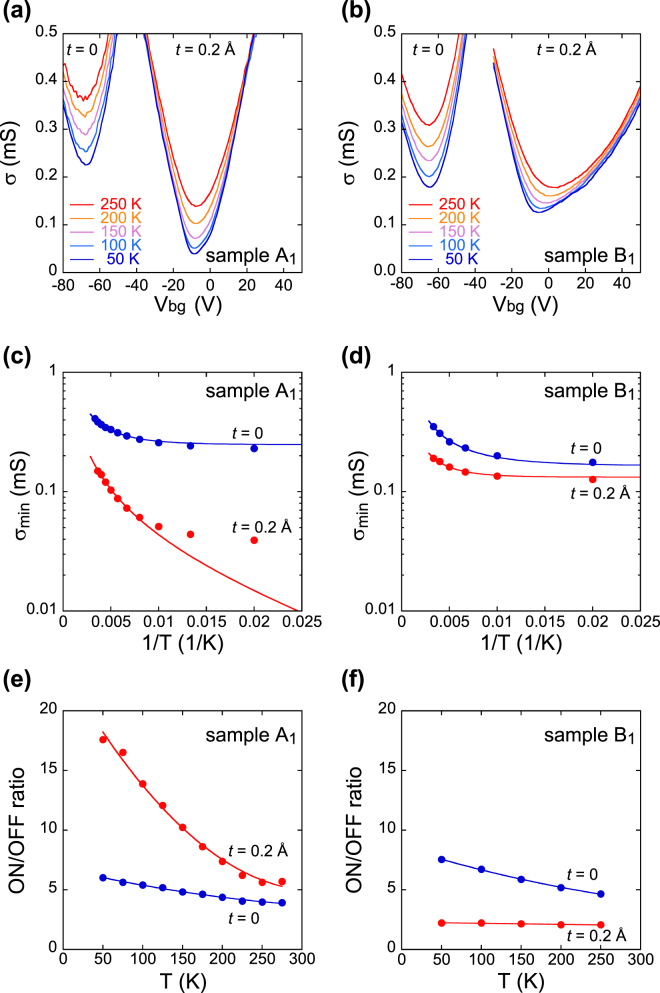
Comparison of altered transport properties after F_4_TCNQ deposition. (**a**,**b**) Conductivity as a function of bottom gate voltage and its temperature dependence. Conductivity curves are shown for *t* = 0 and *t* = 0.2 Å; *t* = 0 refers to ‘before deposition of F_4_TCNQ’, while *t* = 0.2 Å to ‘after deposition of 0.2 Å thick F_4_TCNQ’. The gate voltage was swept from the negative to the positive value, with no hysteresis observed for either sweep direction. (**c**,**d**) Arrhenius plots of the minimum conductivity evaluated from (**a**) and (**b**). The solid lines are fitting curves using equation (2). (**e**,**f**) Temperature dependence of ON/OFF ratio is shown for *t* = 0 and *t* = 0.2 Å. The ON/OFF ratio is defined as *σ*(*V*_n_ + 45 V)/*σ*(*V*_n_), and evaluated from (**a** and **b**). The solid lines are guides to the eye. All graphs in (**a**,**c** and **e**) are based on sample A_1_, and those in (**b**,**d** and **f**) are based on sample B_1_.

A total of four devices (called samples A_1_, A_2_, B_1_, and B_2_) were prepared with the same experimental procedure, but samples A_1_ and A_2_ (called group A), and samples B_1_ and B_2_ (called group B) showed substantially different behavior. Results obtained from samples A_1_ and B_1_ are compared in Fig. 2. Figure 2a,b show the temperature dependence of *σ*(*V*_bg_) before and after the deposition of F_4_TCNQ molecules. In Fig. 2a,b, the charge neutrality point *V*_n_ was initially situated around −65 V. Since *V*_n_ is observed at around 0 V in BLG devices prepared on conventional SiO_2_/Si substrates, the shift of *V*_n_, *ΔV*_n_ ~ −65 V is due to electron accumulation in BLG from NH_2_-SAMs. As shown in Fig. 2a,b, *V*_n_ shifted to around 0 V after the deposition of F_4_TCNQ molecules. This shift *ΔV*_n_ ~ 65 V indicates hole accumulation by F_4_TCNQ molecules in turn. The electron and hole density transferred from each of the molecules is estimated to be *C*_o_|*ΔV*_n_|/*e* ~ 5 × 10^12^ cm^−2^, where *C*_o_ (=11.5 nF cm^−2^) is the capacitance of a 300-nm thick SiO_2_ dielectric. Although the *σ*(*V*_bg_) curves of samples A_1_ and B_1_ were similar to each other before the deposition, they showed a distinct difference after the deposition: the *σ*(*V*_bg_) of sample A_1_ exhibited a sharp dip, while that of sample B_1_ was broadened. This phenomenon is discussed later.

The band structure at the charge neutrality point should be sensitively reflected in the minimum conductivity, *σ*_min_ (=*σ*(*V*_n_)). In Fig. 2c,d, *σ*_min_ is plotted on a logarithmic scale as a function of inverse temperature, 1/*T*, for samples A_1_ and B_1_. When the band gap, *δ*, opens, *σ*_min_ should be limited by the carrier density thermally excited, $n(T)=\exp (-\delta /2{k}_{{\rm{B}}}T)$. Thus, the band gap, if produced, is verified by the enhancement of the slope of the plots in Fig. 2c,d. The slope in the high *T* range increased in sample A_1_ after the deposition, while it decreased slightly in sample B_1_. This result demonstrates the critical difference between the two samples: sample A_1_ exhibited a band gap, but sample B_1_ did not, after F_4_TCNQ deposition.

The difference should also be confirmed by that of ON/OFF ratio between two samples. Figure 2e,f show the temperature dependence of ON/OFF ratio before and after the deposition of F_4_TCNQ molecules. After the deposition, the ON/OFF ratio increased in sample A_1_, while it decreased in sample B_1_. This result can be well explained by different band structure between two samples. The opening of the band gap in sample A_1_ reduced OFF current to enhance the ON/OFF ratio. This enhancement becomes more prominent at low temperature because of the reduction of carrier density excited at OFF state. On the other hand, the ON/OFF ratio in sample B_1_ decreased and did not depend on temperature after the F_4_TCNQ deposition, which indicates no band gap.

### Evaluation of a band gap or band overlap

To evaluate band parameters such as a band gap or band overlap numerically, the temperature dependence of the carrier density, *n*(*T*), was discussed in detail because *σ*_min_ is related to *n*(*T*) by the equation, *σ*_min_(*T*) = *n*(*T*)*eμ*_n_. Here, the carrier mobility at the charge neutrality point, *μ*_n_, is assumed to be constant because the field-effect mobility evaluated from ${\mu }_{{\rm{e}}}=\frac{1}{{C}_{{\rm{O}}}}\frac{d\sigma }{d{V}_{{\rm{b}}{\rm{g}}}}$ at electron regimes was independent of *T* (see Supplementary Fig. S1). Adding up hole and electron density, *n*(*T*) is expressed as

1 $$n(T)={\int }_{-\infty }^{\infty }{D}_{{\rm{h}}}(E)[1-f(E)]dE\,+{\int }_{-\infty }^{\infty }{D}_{{\rm{e}}}(E)f(E)dE.$$

Here, *D*_h_(*E*) and *D*_e_(*E*) are the density of states for holes and electrons in BLG. $f(E)\equiv {[1+\exp (\frac{E-{E}_{{\rm{F}}}}{{k}_{{\rm{B}}}T})]}^{-1}$ is the Fermi-Dirac distribution function, *E*_F_ is the Fermi energy, set to be 0 at the charge neutrality point, and *k*_B_ is the Boltzmann constant. Assuming constant *D*_h_(*E*) and *D*_e_(*E*) of BLG for simplicity:

$${D}_{{\rm{h}}}(E)=\{\begin{array}{c}D,\quad E\le -\delta /2\\ 0,\quad E > -\delta /2\end{array}\,{\rm{a}}{\rm{n}}{\rm{d}}\,{D}_{{\rm{e}}}(E)=\{\begin{array}{c}0,\quad E < \delta /2\\ D,\quad E\ge \delta /2\end{array},$$

the minimum conductivity is obtained from

2 $${\sigma }_{{\rm{\min }}}(T)=2e{\mu }_{{\rm{n}}}D{k}_{{\rm{B}}}T\,\mathrm{ln}[1+\exp (-\delta /2{k}_{{\rm{B}}}T)].$$

Here, a positive *δ* means a band gap with energy of *δ*, while negative *δ* corresponds to a band overlap with energy of −*δ*. In the low *T* limit, equation (2) is reduced to ${\sigma }_{min}\sim 2e{\mu }_{{\rm{n}}}D{k}_{{\rm{B}}}T\exp (-\frac{\delta }{2{k}_{{\rm{B}}}T})$ for *δ* > 0 and ${\sigma }_{\min } \sim e{\mu }_{{\rm{n}}}D(-\delta )$ for *δ* < 0. A negative *δ* can also be the result of Fermi level broadening due to local potential fluctuation^38, 39^, as discussed later. The experimental data in the range of 125 K ≤ *T* ≤ 275 K in Fig. 2c,d were fitted with equation (2), and the results are shown with solid lines. The fitting lines show good agreement with experimental data over the entire *T* range except for the data at *t* = 0.2 Å of sample A_1_. The discrepancy at *t* = 0.2 Å of sample A_1_ in the low *T* range is ascribed to the residual conductance due to the variable range hopping, which is noticeable in the presence of a band gap^22–24^. Actually, the *δ* value of sample A_1_ increases from −34 to 8.1 meV, indicating the generation of a band gap. On the other hand, the *δ* of sample B_1_ decreases from −25 to −37 meV, indicating the enhancement of band overlap. Thus, samples A_1_ and B_1_ were affected differently by the deposition of F_4_TCNQ molecules.

**Figure 3:**
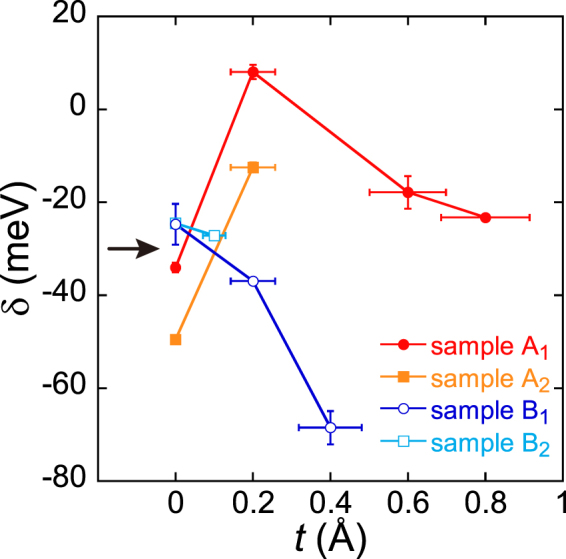
Band parameter as a function of F_4_TCNQ thickness. The band parameter *δ* was evaluated using equation (2). Positive and negative *δ* value means the separation and overlap of the valence and conduction bands, respectively. At *t* = 0, all *δ* was negative because of the spatial potential variation (see the main text and Fig. 4a). After the initial deposition of F_4_TCNQ, *δ* increased in two samples (samples A_1_ and A_2_ are called group A, solid symbols). The positive *δ* (*t* = 0.2 Å) in sample A_1_ means the opening of a band gap. The *δ* value in sample A_2_ was also increased but still negative, indicating the decrease in the band overlap or the trend of the band gap opening. In contrast, *δ* decreased in two samples (samples B_1_ and B_2_ called group B, open symbols), indicating the enhancement of the band overlap. With further deposition of F_4_TCNQ, *δ* for both samples A_1_ and B_1_ decreased. The arrow indicates the boundary at *δ*(0) ~ −30 meV that distinguishes groups A and B.

The *σ* *− V*_bg_ curves and *σ*_min_ − 1/*T* plots for samples A_2_ and B_2_ are shown in Supplementary Fig. S2. The values *δ* for samples A_2_ and B_2_ were evaluated from the analysis using equation (2). In Fig. 3, *δ* values for all four samples are plotted as a function of *t*. After the first deposition of F_4_TCNQ, *δ* increased for both devices in group A, indicated by the closed symbols, while *δ* decreased for both in group B, indicated by the open symbols. A band gap tends to open in group A, but it does not in group B. With increasing *t* furthermore, *δ* decreased to a negative value even for sample A_1_. To summarize the experimental results, electron and hole doped BLG (or BLG sandwiched by electron-donor SAMs and electron-acceptor molecules) did not always exhibit a band gap even under identical preparation and measurement conditions. This result seems to correspond to previous conflicting reports about the opening of a band gap.

## Discussion

**Figure 4:**
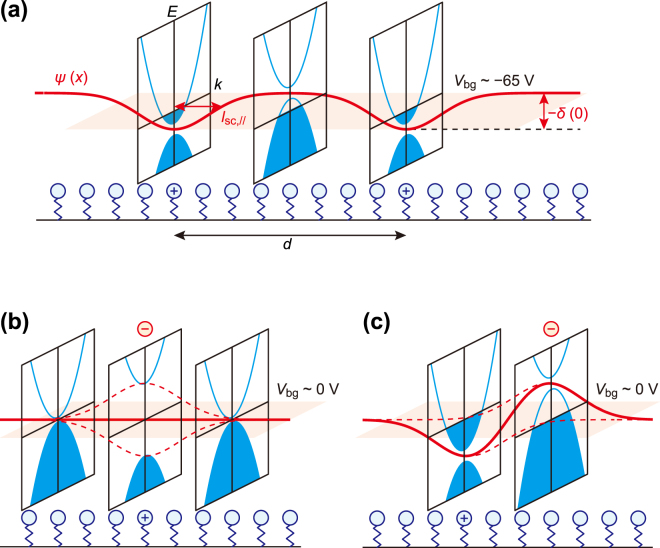
Potential profiles depending on dopant arrangement. (**a**) The red line indicates the spatial variation of the potential energy *ψ*(*x*) in BLG on NH_2_-SAMs. NH_2_^+^- and NH_2_-AS are distinguished by ⊕ and ○. *ψ*(*x*) shows the minimum on NH_2_^+^-AS and changes spatially in accord with the characteristic screening length *l*_SC,//_. The blue curves show the band structure *E*(*k*) modified by *ψ*(*x*). The horizontal plane indicates the Fermi energy *E*_F_ at the charge neutrality point around *V*_bg_ ~ −65 V. At a position far from NH_2_^+^-AS, *i.e*., the position corresponding to the middle energy diagram, *ψ*(*x*) is mainly affected by *V*_bg_ ~ −65 V. The negative *V*_bg_ produces a downward electric field to open the band gap. Nonetheless, *E*_F_ is located at the valence band, producing a hole puddle. However, at a position just above NH_2_^+^-AS, *i.e*., the position corresponding to the left or right energy diagram, the effect of NH_2_^+^-AS is larger than that of *V*_bg_, producing an upward electric field. In this case, *E*_F_ is located in the conduction band, producing an electron puddle. In this way, both hole and electron puddles contribute to carrier transport at the charge neutrality point. (**b**) NH_2_^+^-AS and F_4_TCNQ^−^ molecules, denoted by ⊕ and $\ominus$, are aligned in the normal direction. The horizontal plane indicates *E*_F_ at the charge neutrality point around *V*_bg_ ~ 0 V. Two red dashed curves show the potential energy produced by each molecule, which mutually cancel. In this case, the perpendicular electric field produces the band gap, which directly affects the transport property at the charge neutrality point. **(c)** NH_2_^+^-AS and F_4_TCNQ^−^ molecules are not aligned. The horizontal plane indicates *E*_F_ at the charge neutrality point around *V*_bg_ ~ 0 V. When the lateral spacing of two dopants is larger than *l*_SC,//_, the potential energy produced by each molecule is not cancelled but varies with location. In this case, the transport property is determined by electron and hole puddles as in (**a**). The different arrangement in (**b** and **c**) originates in the magnitude of potential variation and the screening length *l*_SC,//_.

We first consider why the behavior of *δ*(*t*) is different in groups A and B. It is worth noting that the *δ* before the deposition of F_4_TCNQ molecules, *δ*(0), is smaller in group A than B; the *δ*(0) values of group A are below −30 meV, while those of group B are above −30 meV (Fig. 3). The values of *δ*(0) are negative for all samples, which is ascribed to the potential irregularity induced by charged molecules. The molecular density of SAMs^33^, *i.e*., the number of NH_2_-alkylsilane (NH_2_-AS) per unit area is typically (1–2) × 10^14^ cm^−2^, while the electron density transferred from NH_2_-SAMs is estimated to be 5 × 10^12^ cm^−2^ as described before. This means that only one out of 20–40 NH_2_-ASs donates an electron to the BLG. Consequently, two kinds of molecules are present in NH_2_-SAMs. One is an ionized molecule that has donated an electron, referred to as NH_2_^+^-alkylsilane (NH_2_^+^-AS). The other is a neutral molecule which does not donate an electron, referred to as NH_2_-AS. The potential energy of an electron in BLG is lower near an NH_2_^+^-AS than that near an NH_2_-AS. In this case, both the electrons and holes can contribute to the electric transport even at the charge neutrality point, as seen from Fig. 4a. This situation means that the conduction and valence bands overlap. Thus, the value of −*δ*(0) shown in Fig. 4a indicates the magnitude of the potential variability in BLG, which is larger in group A than group B (−*δ*(0) for A > −*δ*(0) for B).

Note that the potential fluctuation caused by NH_2_-SAMs affects the top surface of the BLG. This can be understood by considering that the potential energy varies with the characteristic screening length of BLG in the intralayer and interlayer directions. The intralayer screening length, *l*_SC,//_, is estimated to be *l*_SC,//_ ≡*κħ* ^2^/(*g*_s_ *g*_v_ *me* ^2^) ~ 1.1 nm^40^, where *κ* ~ 2.5 is the average background dielectric constant, *ħ* is the Planck’s constant divided by 2*π*, *g*_s_ = *g*_v_ = 2 is the spin and valley degeneracy, and *m* = 0.03*m*_e_ is the effective mass in BLG^40^. The length of *l*_SC,//_ is less than the average distance between NH_2_^+^-ASs, $d\sim 2\sqrt{e/(\pi {C}_{{\rm{O}}}|{\rm{\Delta }}{V}_{{\rm{n}}}|)}\sim 5.2\,\,{\rm{n}}{\rm{m}}$ (Fig. 4a). Since *l*_SC,//_ is shorter than *d*, the spatially inhomogeneous potential is created in BLG as shown in Fig. 4a. On the other hand, the interlayer screening length, *l*_SC,⊥_, of few-layer graphene exceeds 2 layers around the charge neutrality point^41–43^. Comparing the screening length, *l*_SC,⊥_, and the thickness of BLG shows that the potential fluctuation due to the bottom dopant is not completely screened by BLG but affects the surface of the top layer. This can influence the arrangement of F_4_TCNQ molecules deposited and thus change the collective electronic properties of the entire construct.

Figure 4b,c illustrate the potential profiles of BLG, which depend on the alignment of the NH_2_^+^-AS and F_4_TCNQ^−^ molecules. When the potential fluctuation, −*δ*(0), is large enough, as in group A, an F_4_TCNQ molecule, which is ionized to F_4_TCNQ^−^ after accepting an electron, may be trapped by the NH_2_^+^-AS as shown in Fig. 4b. Since the two dopants are aligned in the normal direction, the perpendicular electric field is generated to open the band gap. However, when the potential fluctuation, −*δ*(0), is small, as in group B, F_4_TCNQ molecules can be adsorbed anywhere without restriction, as in Fig. 4c. In this case, the average lateral distance between randomly deposited NH_2_^+^-AS and F_4_TCNQ^−^ molecules is estimated to be $d/\sqrt{2}\sim 3.7\,{\rm{n}}{\rm{m}}$, which still exceeds the intralayer screening length, *l*_SC,//_. Consequently, the potential fluctuation is further enhanced as seen from Fig. 4c, which prevents the opening of a band gap because of the overlapping of potentials as illustrated in Fig. 4c. The difference in molecular alignments shown in Fig. 4b,c depends on whether the F_4_TCNQ^−^ molecule is trapped by the potential drop at the NH_2_^+^-AS. The threshold energy for the trapping of the molecules in the potential fluctuation corresponds to thermal energy at room temperature, 26 meV. This estimation shows a good agreement with the experimental boundary around 30 meV (−*δ*(0) for A > 30 meV, −*δ*(0) for B < 30 meV) shown in Fig. 3, strongly supporting our discussion.

Thus, the alignment of the NH_2_^+^-AS and F_4_TCNQ^−^ molecules via BLG is required for band gap opening. The random deposition of F_4_TCNQ molecules increases the potential variability, thereby enhancing the potential scattering. The decrease in carrier mobility due to scattering is indicated by the broadening of *σ*(*V*_bg_) in Fig. 2b. In order to open the maximum energy gap, not only the alignment but also the number of F_4_TCNQ molecules must be optimized to compensate for the effect of NH_2_^+^-AS. As shown in Fig. 3, the excess deposition of F_4_TCNQ increases −*δ* even in sample A_1_, meaning that F_4_TCNQ molecules in excess of the optimal value only enhance the potential variability.

To generalize our claim and ensure the reproducibility of results, we additionally carried out transport measurement of two devices (samples C_1_ and C_2_) which were prepared on SiO_2_/Si substrate without NH_2_-SAMs. As shown in Supplementary Fig. S3, the *δ*(0) value of sample C_1_ was around −30 meV, and that of sample C_2_ was less than −30 meV. The *δ* value of both samples slightly increased after the F_4_TCNQ deposition, indicating the trend of the band gap opening. The data obtained from samples C_1_ and C_2_ seem to follow the scenario derived from other samples (A and B) prepared on NH_2_-SAMs, supporting our conclusion that the opening of the band gap is determined by *δ*(0), *i.e*., the potential variation caused by charged impurities on the substrate governs the electronic property after the deposition of F_4_TCNQ molecules.

Finally, we consider the origin of the different values of *δ*(0) in different devices. Since the devices were prepared in the same manner, some uncontrollable factors may affect *δ*(0). For example, when BLG is transferred onto the NH_2_-SAMs using the micromechanical cleavage technique^34^, the spacing between BLG and NH_2_-SAMs may change depending on devices owing to the flexibility of the alkyl chains in NH_2_-SAMs. In this case, the smaller the spacing, the larger the −*δ*(0). Additional factors originating in the interaction of NH_2_-SAMs and BLG such as corrugation^44^ of the BLG may also affect the potential fluctuation, −*δ*(0). One possible solution to open the band gap in BLG under any conditions is to deposit F_4_TCNQ molecules at low temperature. The molecules will tend to be trapped at NH_2_^+^ sites and thus to promote the band gap opening, since the low thermal energy suppresses molecular motion.

In conclusion, we studied the electronic states of BLG, both surfaces of which were decorated with electron transfer molecules. The temperature dependence of conductivity showed that the band gap was opened in some devices and was not in others, which seems consistent with previous inconsistent results. We have concluded that the difference originates in local potential variations due to dopant molecules. The effect of the potential variation on BLG is significant because of the long interlayer and short intralayer screening length in BLG. Furthermore, our results show an important difference between gating and doping methods. Compared with the gating method, the doping method is more sensitive to the potential irregularities caused by the dopants. In the gating method using a conventional 300-nm thick SiO_2_ dielectric, the effect of local potential variations is averaged out. When very thin gate dielectrics such as an electric-double layer are used, the effect of potential irregularities becomes important even in the gating method. Our study of the microscopic arrangement of molecular dopants and its controllability is significant for the application of an electron-transfer molecule for the ultimate small gate electrode. The findings may open new avenues for the nano-technological development of molecular electronics.

## Methods

### Device preparation

NH_2_-SAMs were prepared on a SiO_2_/Si substrate as in the previous report^33^. Its uniformity was confirmed by measuring the contact angle of a water droplet placed on the NH_2_-SAMs. The contact angle was 81.2(9)°, which was larger than the values reported previously^33^, indicating the uniformity of NH_2_-SAMs. BLG devices on NH_2_-SAMs were prepared by the conventional microfabrication technique using electron beam lithography, Elionix Inc., ELS-S50.

### Measurement

Transport measurement was carried out in a cryogenic probe station that is combined with a chamber for molecular deposition (Riko International LTD, i-series ultrahigh vacuum microprobe and chamber). In advance of the measurement, the devices were annealed at 373 K in a vacuum for over 1 hour in order to eliminate adsorbed atmospheric gases such as H_2_O or O_2_ molecules. Four-terminal conductivity was measured using a semiconductor device analyzer, Agilent B1500A. The devices were transferred from the probe system to the other vacuum chamber, in which F_4_TCNQ molecules were deposited on the devices under a pressure of 10^−6^ Pa.

### Data availability

All data generated or analysed during this study are included in this published article (and its Supplementary Information files).

## Electronic supplementary material

Supplementary Information
