# Supplementary material for: Difference in gating and doping effects on the band gap in bilayer graphene
Source: Sci Rep. 2017 Sep 12;7:11322. doi: 10.1038/s41598-017-11822-9 (PMC5595964; doi:10.1038/s41598-017-11822-9)
Supplement: Supplementary file 1 — Supplementary Information [file 41598_2017_11822_MOESM1_ESM.pdf]

Supplementary Information

Difference in gating and doping effects on a band gap of bilayer graphene

Takaki Uchiyama[1], Hidenori Goto[1*], Hidehiko Akiyoshi[1], Ritsuko Eguchi[1], Takao Nishikawa[2], Hiroshi Osada[3], Yoshihiro Kubozono[1]

[1]*Research Institute for Interdisciplinary Science, Okayama University, Okayama 700-8530, Japan*

[2]*Hanamaki Satellite, Research Center for Industrial Science, Iwate University, Iwate 025-0312, Japan*

[3]*Faculty of Science and Engineering, Iwate University, Iwate 020-8551, Japan*

Contents

S1. Temperatue dependence of field-effect mobility

S2. Transport properties of sample A_2 and B_2

S3. Bilayer graphene devices prepared on SiO_2/Si substrate

S1. Temperatue dependence of field-effect mobility

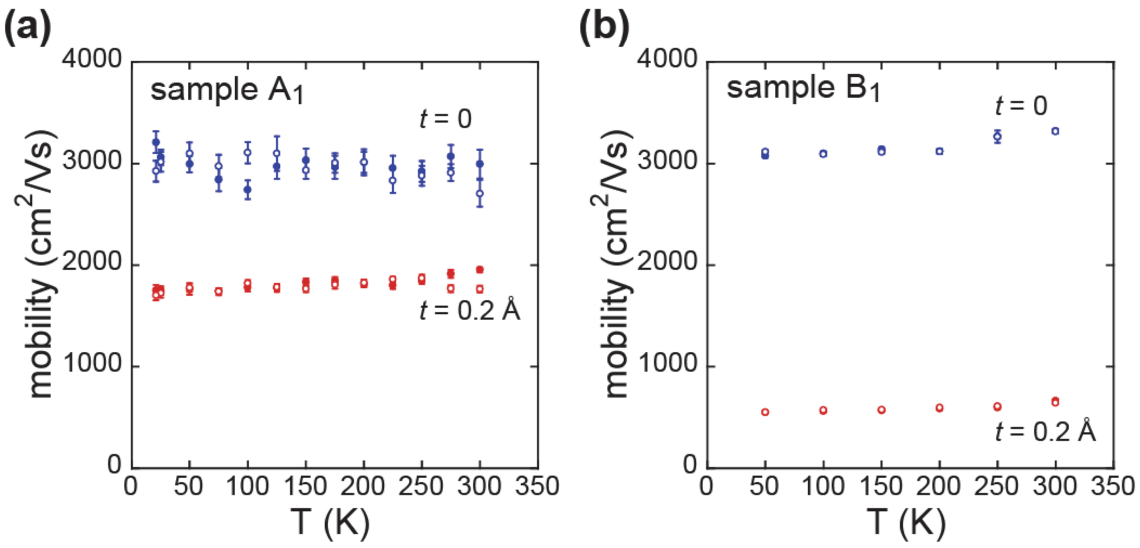

Supplementary Figure S1 | temperature dependence of field-effect mobility. (a,b) field-effect mobility μ_e of (a) sample A_1 and (b) sample B_1 is shown for $t = 0$ (blue symbols) and $t = 0.2$ Å (red symbols). The values of μ_e are evaluated from the relation, $\mu_e = \frac{1}{C_o}\frac{d\sigma}{dV_{bg}}$, in the range of -30 V < V_{bg} < -10 V for $t = 0$ and 30 V < V_{bg} < 50 V for $t = 0.2$ Å. The solid and open symbols correspond to the mobility estimated from positive and negative sweep directions of V_{bg}, respectively.

S2. Transport properties of sample A_2 and B_2

Temperature dependence of $\sigma(V_g)$ curves and Arrhenius plots of the minimum conductivity obtained from sample A_2 and B_2 are shown in Fig. S2. The $\sigma(V_g)$ curves of each sample showed that the charge neutrality point shifted from the −60 ~ −70 V to −10 ~ −20 V after F_4TCNQ deposition, which confirms the electron doping from NH_2-SAMs and hole doping from F_4TCNQ molecules. The Arrhenius plots of the minimum conductivity clearly showed distinguished features; sample A_2 showed the trend of band gap, while sample B_2 did not.

Fitting parameters obtained from transport measurements are summarized in Table S1. Here, δ and $\mu_n D$ were obtained from the fitting with equation (2) in the main text, and field-effect mobility μ_e was estimated in the electron regime. Note that both samples A_1 and A_2 exhibited the band gap, although they showed very different values of $\mu_n D$ and μ_e before F_4TCNQ deposition. This suggests that the values of $\mu_n D$ and μ_e do not relate to the band gap opening. As seen from Table S1, δ (0) is the important parameter to distinguish groups A and B.

Finally, we compare theoretical and experimental values of D. Based on the tight binding model for bilayer graphene, D is calculated to be 3.0×10^{13} $eV^{-1}cm^{-2}$ at the charge neutrality points. The value of μ_n may be different from μ_e, and impurities states due to adsorbed molecules may increase D; nevertheless experimental values of $\mu_n D/\mu_e$ are in good agreement with the calculated value, which confirms the validity of our analysis.

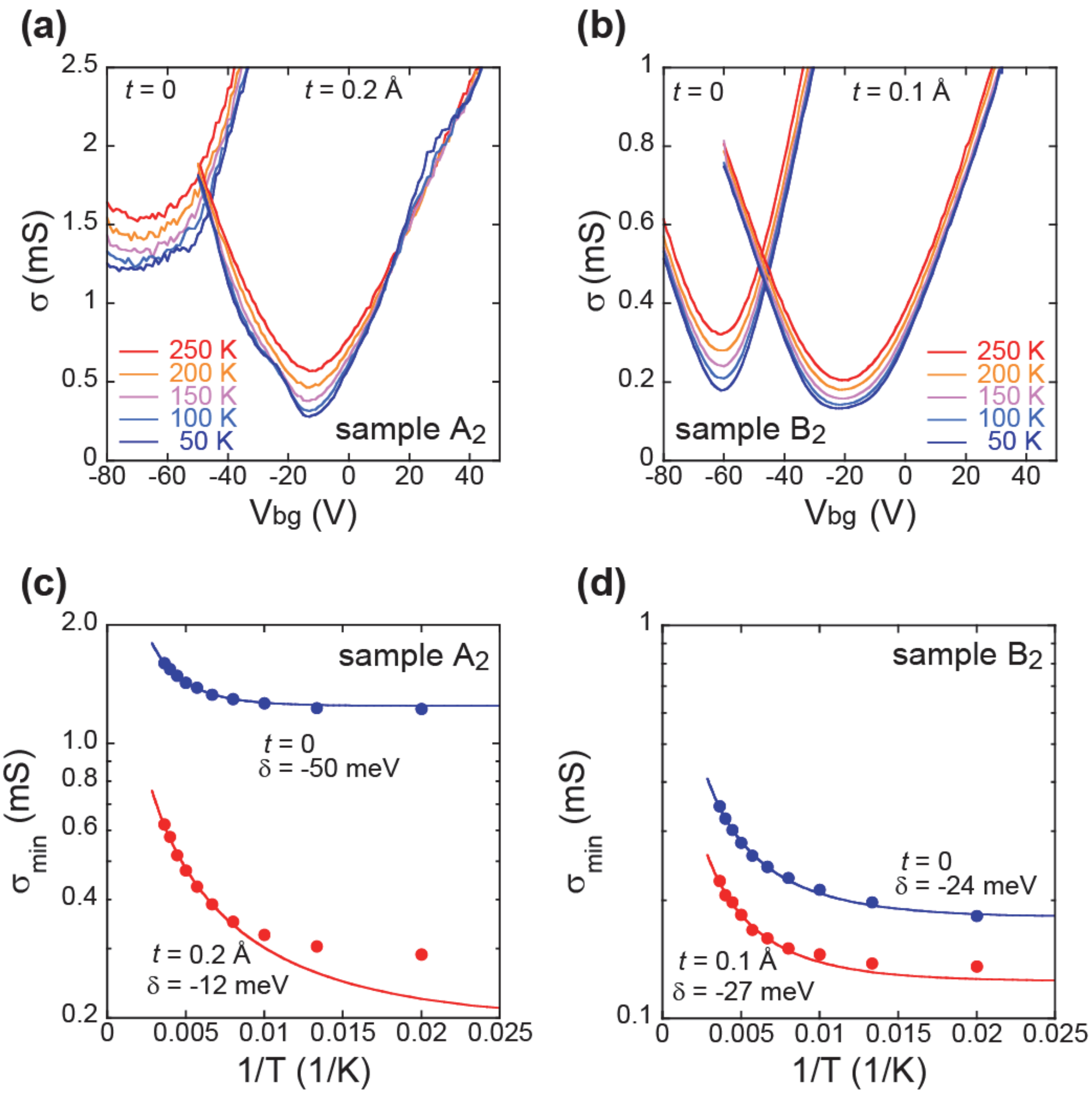

Supplementary Figure S2 | Transport properties of sample A_2 and B_2 before and after F_4TCNQ deposition. (a,b) Conductivity as a function of bottom gate voltage and its temperature dependence. Conductivity curves are shown for *t* = 0 and 0.2 Å (sample A_2), and for *t* = 0 and 0.1 Å (sample B_2). The gate voltage was swept from the negative to the positive value, with no hysteresis observed for either sweep direction. **(c,d)** Arrhenius plots of the minimum conductivity evaluated from (a) and (b). The solid lines are fitting curves using equation (2) in the main text. All graphs in (a) and (c) are based on sample A_2, and those in (b) and (d) are based on sample B_2.

Supplementary Table S1 | Fitting parameters obtained from transport measurements

Sample	δ (meV)	$\mu_n D$ (10^{16} $V^{-1}s^{-1}eV^{-1}$)	μ_e (10^3 $cm^2V^{-1}s^{-1}$)	$\mu_n D/\mu_e$ (10^{13} $V^{-1}cm^{-2}$)
A_1 (t = 0)	−34	4.6	3.0	1.5
A_1 (t = 0.2 Å)	8.1	3.3	1.8	1.8
A_2 (t = 0)	−50	16	5.8	2.8
A_2 (t = 0.2 Å)	−12	9.8	3.5	2.8
B_1 (t = 0)	−25	4.3	3.1	1.4
B_1 (t = 0.2 Å)	−37	2.1	0.6	3.5
B_2 (t = 0)	−24	4.6	3.3	1.4
B_2 (t = 0.1 Å)	−27	2.9	2.1	1.4

S3. Bilayer graphene devices prepared on SiO_2/Si substrates

We prepared bilayer graphene devices on SiO_2/Si substrates without using NH_2-SAMs (samples C_1 and C_2), and studied the change in the transport properties before and after F_4TCNQ deposition in the same way described in the main text. The δ values for sample C_1 and C_2 were evaluated using equation (2) in the main text, and plotted in Fig. S3.

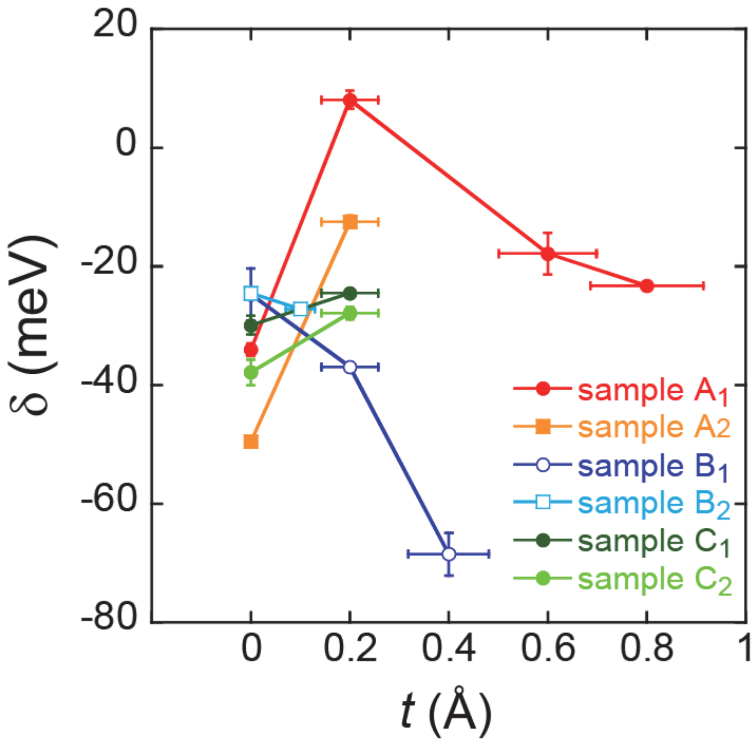

Supplementary Figure S3 | Band parameter as a function of F_4TCNQ thickness. The data obtained from samples C_1 and C_2, which were prepared on SiO_2/Si substrate, are added into Fig. 3 in the main text.
